# Hyponatremia Associated with Congestive Heart Failure: Involvement of Vasopressin and Efficacy of Vasopressin Receptor Antagonists

**DOI:** 10.3390/jcm12041482

**Published:** 2023-02-13

**Authors:** San-e Ishikawa, Hiroshi Funayama

**Affiliations:** 1 Department of Medicine, Jichi Medical University, Shimotsuke 329-0498, Tochigi, Japan; 2 Department of Cardiology, Jichi Medical University, Shimotsuke 329-0498, Tochigi, Japan

**Keywords:** hyponatremia, heart failure, acute myocardial infarction, prognosis, arginine vasopressin (AVP), baroreceptor, circulatory blood volume, AVP receptor antagonists

## Abstract

Hyponatremia is frequently found in patients with congestive heart failure. A reduction in effective circulatory blood volume in a volume-expanded patient with decreased cardiac output is linked to a baroreceptor-mediated non-osmotic release of arginine vasopressin (AVP). The increased production of AVP and salt and water retention in the proximal and distal tubules of the kidney by humoral, hemodynamic, and neural mechanisms increase circulatory blood volume and contribute to hyponatremia. Recent studies have indicated that hyponatremia predicts the short-term and long-term prognosis of heart failure by increasing cardiac death and rehospitalization. In addition, the early development of hyponatremia in acute myocardial infarction also predicts the long-term prognosis of worsening heart failure. AVP V2 receptor antagonism may relieve water retention, but it is unknown whether the V2 receptor inhibitor, tolvaptan, improves the long-term prognosis of congestive heart failure. The newly identified natriuretic factor in renal salt wasting has the potential of improving clinical outcomes when combined with a distal diuretic.

## 1. Introduction

Hyponatremia is linked to reduced myocardial contractility, which leads to a complicated set of pathophysiological events that increase circulatory blood volume, edema formation, characteristic multiorgan derangements, and the clinical manifestation of congestive heart failure (CHF). The increase in total body sodium (Na) and water volume in CHF is due to a combination of a baroreceptor-mediated non-osmolar increase in arginine vasopressin (AVP) release and impaired renal sodium (Na) and water excretion via the activation of the sympathetic nervous system, increased renin, angiotensin and aldosterone production, and hemodynamic alterations. In this review, we discuss the pathophysiologic mechanisms that increase total body Na and water, the induction of hyponatremia, how hyponatremia contributes to short-term and long-term outcomes in CHF and therapy with AVP V2 receptor antagonist, and a newly identified natriuretic protein.

### 1.1. Hyponatremia in Congestive Heart Failure

Clinical and epidemiological studies have found hyponatremia to be a common phenomenon in CHF. As described in greater detail below, a reduction in “effective” circulatory blood volume, despite an increased circulatory blood volume, is closely linked to an exaggerated release of endogenous hormones and the development of hyponatremia [1]. Gheorghiade et al. [2] found hyponatremia, defined as serum Na less than 135 mmol/L, to be present in 19.7% of 48,612 patients with CHF from a total of 259 hospitals across all regions of the United States between 2003 and 2004. The subjects were elderly with a mean age of 73 years, including 52% women and 74% Caucasian participants. The mean admission serum sodium (Na) was 138 ± 5 mmol/L. There were no differences in age, gender, etiology, diabetes, ejection fraction, or symptoms of congestion between the hyponatremic patients and patients with normal serum Na levels [2]. Similar findings were obtained in patients with early-phase ST elevation in acute myocardial infarction [3,4]. Tada et al. [5] found that 20.7% of patients with acute myocardial infarction had serum Na levels of less than 136 mmol/L 72 h after admission (early development of hyponatremia). Other studies by Goldberg et al. [6], Klopotowski et al. [7], and Singla et al. [8] also demonstrated a relationship between hyponatremia and long-term mortality and progression to CHF in survivors of acute ST-elevation myocardial infarction. Thus, early development of hyponatremia in the setting of acute myocardial infarction appears to be a prognostic indicator of incipient CHF and long-term mortality.

### 1.2. Pathogenesis of Exaggerated Release of Arginine Vasopressin

Increases in plasma AVP, renin activity, aldosterone, and norepinephrine have been demonstrated in several animal models of low-output and high-output cardiac failure and in CHF in humans [9,10]. The non-suppressible, non-osmotic release of AVP was prominently related to an increased expression of AVP mRNA in the hypothalamus of rats after induction of CHF by ligating coronary arteries [11]. The increase in free water excretion after the administration of an AVP V2 receptor antagonist has elucidated the important role of AVP in the impairment of renal water excretion in CHF [12,13].

**Figure 1:**
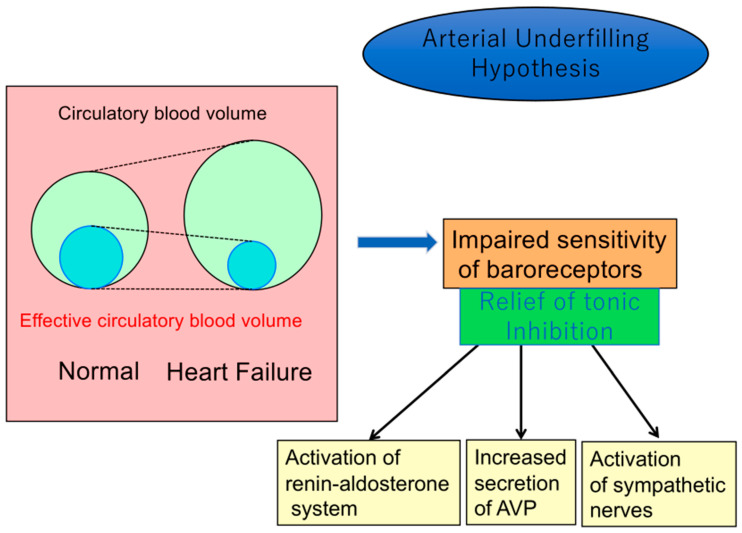
Arterial underfilling hypothesis for dysregulation of baroreceptor-mediated hormonal release in congestive heart failure.

Renal Na and water excretions are regulated, in part, by alterations in the arterial circulation that are determined by cardiac output and peripheral vascular resistance. Several baroreceptors on the high-pressure side of the circulation in the carotid sinus, left atrium, aortic arch, and renal afferent arterioles can sense arterial underfilling, which alters their sensitivity. An altered sensitivity of baroreceptors occurs when there is a reduction in systemic arterial pressure, cardiac stroke volume, renal perfusion, or peripheral vascular resistance. A decrease in “effective” circulatory blood volume, promoted by low cardiac output, impairs the sensitivity of baroreceptors. This altered baroreceptor sensitivity could relieve the “tonic inhibition” on afferent vagal nerve parasympathetic pathways of hormonal synthesis to induce a non-osmolar increase in AVP secretion by the hypothalamo-neurohypophyseal system [14,15,16]. In addition, a decrease in effective circulatory blood volume activates the sympathetic nervous system, the renin–angiotensin–aldosterone system, as well as the baroreceptor-mediated non-osmotic release of AVP (Figure 1). However, how baroreceptors sense the reduction in effective circulatory blood volume in heart failure remains to be determined.

**Figure 2:**
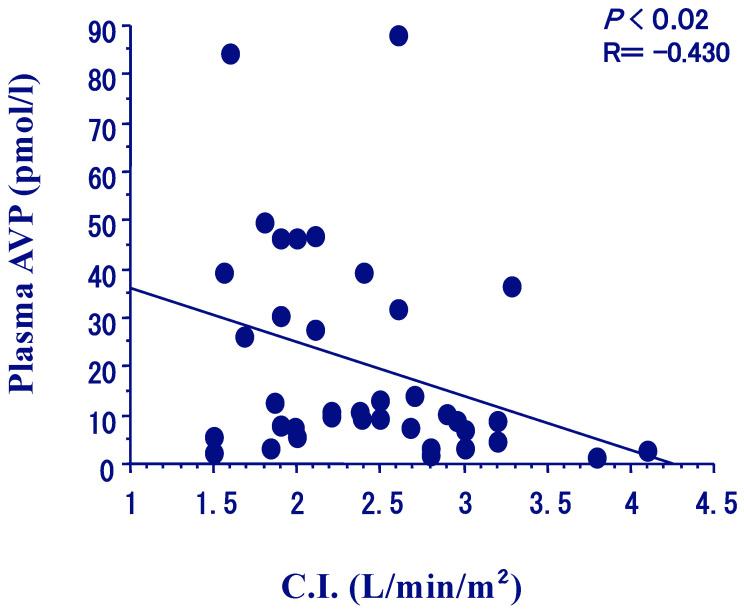
Relationship of plasma AVP levels with cardiac index (CI) in patients with congestive heart failure.

The hydro-osmotic action of AVP in the kidneys is mediated through AVP V2 receptors on the basolateral membranes of renal collecting duct cells. The receptor binding with AVP activates adenylate cyclase to produce cyclic adenosine monophosphate (cAMP), which leads to the phosphorylation of cAMP-dependent protein kinase (PKA). The cAMP-PKA pathway mediates the formation of aquaporin 2 (AQP2) water channels, which fuse and translocate to the apical plasma membrane (short-term regulation), increase AQP2 transcription and protein synthesis (long-term regulation) [17], and expose the water channels to an osmotic gradient that transports water from the distal tubular lumen urine to the medullary interstitium. Approximately 3% of AQP2 channels are excreted in the urine as urine AQP2 (UAQP2) [18]. UAQP2 exists in soluble and membrane-bound forms that have a positive correlation with plasma AVP levels in normal humans [19,20,21]. We determined the plasma AVP levels and UAQP2 in 65 patients with class I to IV New York Heart Association (NYHA) classification CHF [22]. Plasma AVP and UAQP2 increased gradually with worsening CHF, and there was a negative correlation between plasma AVP levels and the cardiac index or severity of CHF. The results of Swan-Ganz catheterization revealed that the cardiac index reflected the severity of CHF with a negative correlation between plasma AVP levels and the cardiac index (Figure 2).

The decrease in effective circulatory blood volume decreases the glomerular filtration rate (GFR) and activates the sympathetic nervous and renin–angiotensin–aldosterone systems [14,15]. Increased renin synthesis by the juxtaglomerular cells of the renal afferent and efferent arterioles converts angiotensinogen to angiotensin-I and ultimately to angiotensin II. Angiotensin II contributes to the reduction in GFR by vasoconstricting the renal arteries, increases proximal tubular Na and water reabsorption, and decreases the distal delivery of Na and water. Angiotensin II also stimulates the secretion of aldosterone, which reduces Na and water excretion in the final urine by increasing distal tubular Na reabsorption. Bichet et al. [23] demonstrated that angiotensin-converting enzyme inhibitors and α1-adrenergic action reduced cardiac afterload, increased cardiac output, and improved renal water excretion in patients with NYHA class III and IV CHF. The increased activity of the renin–angiotensin–aldosterone and sympathetic nervous systems and hemodynamic adjustments, however, override these effects by increasing the net renal Na and water reabsorption to maintain the fluid overload.

### 1.3. Hyponatremia as a Prognostic Factor

Studies have investigated the prognostic impact of hyponatremia in patients with CHF [2,4,24,25]. Gheorghiade et al. [2] reported that patients with left ventricular systolic dysfunction with an admission serum Na of less than 135 mmol/L were associated with poor short-term and long-term prognoses for the length of hospital stay and in-hospital mortality compared with those without hyponatremia. There was also an increase in long-term rehospitalization and cardiac mortality in hyponatremic patients with recurrent CHF [2,4,24]. In addition, the OPIMIZE-HF study demonstrated that in-hospital and follow-up mortality risks in patients with left ventricular systolic dysfunction increased by 19.5 and 10.9%, respectively, for each 3 mmol/L decrease in serum Na below 140 mmol/L after adjustment for other prognostic variables [2]. The risks for these hyponatremic patients were approximately twice as high as those for patients with normal to high serum Na levels. Thus, admission serum Na levels are independent predictors of in-hospital and post-discharge mortality and rehospitalization rates.

**Figure 3:**
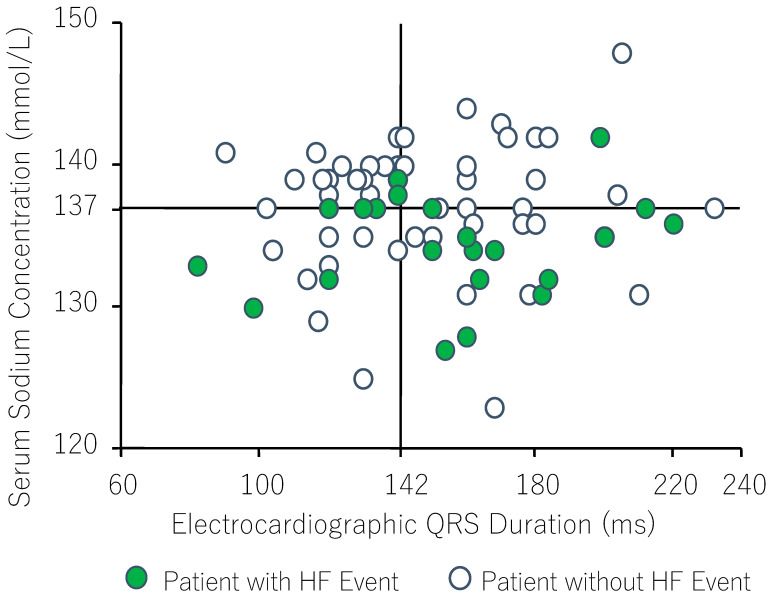
Distribution of serum Na levels and electrocardiographic QRS duration in patients with congestive heart failure. Each solid line shows the median value. Heart failure events occurred more often in hyponatremic patients, especially those with widened QRS duration. Cited from Ref. [26].

Furthermore, we reported that hyponatremia only affected long-term prognosis in patients with heart failure who received cardiac resynchronization therapy (CRT) [26]. Twenty-two of seventy-seven patients (29%) aged 24 to 82 years with stage II, III, and IV NYHA heart failure and left ventricular ejection fractions below 35% who were treated by CRT for a median of 601 days had worsening heart failure. Univariate analysis by the Cox proportional hazards model revealed that young age, NYHA class IV, high plasma BNP, high serum creatinine, low serum Na, and low mean blood pressure were associated with worsening heart failure. In the multivariate analysis, only hyponatremia was associated with the occurrence of heart failure (Hazard ratio 0.82, *p* = 0.034). Moreover, patients with heart failure had higher plasma AVP levels and hyponatremia with electrocardiographic evidence of an increased QRS interval on admission compared with those without heart failure (Figure 3). Similar findings were obtained by other investigators [4,24]. It thus appears that a reduction in myocardial contractility creates a vicious cycle of arterial underfilling and the activation of pathophysiological systems that lead to increased Na and water retention to increase total body Na and water and edema formation and activate a baroreceptor-mediated non-osmotic increase in AVP to induce hyponatremia.

**Figure 4:**
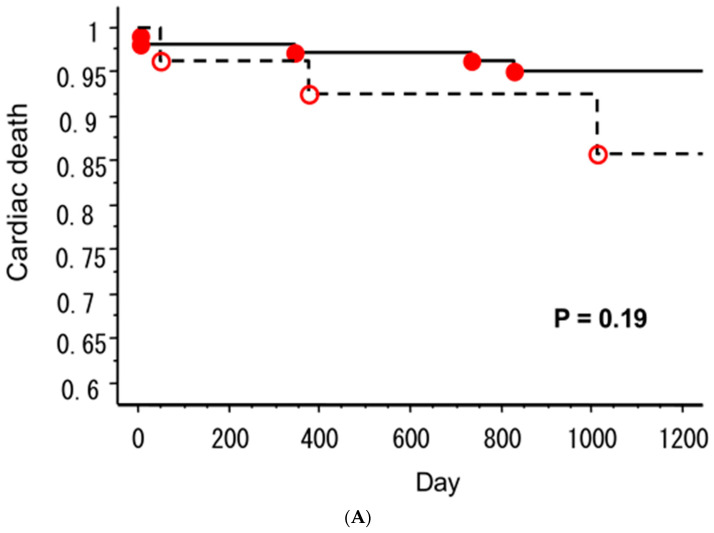

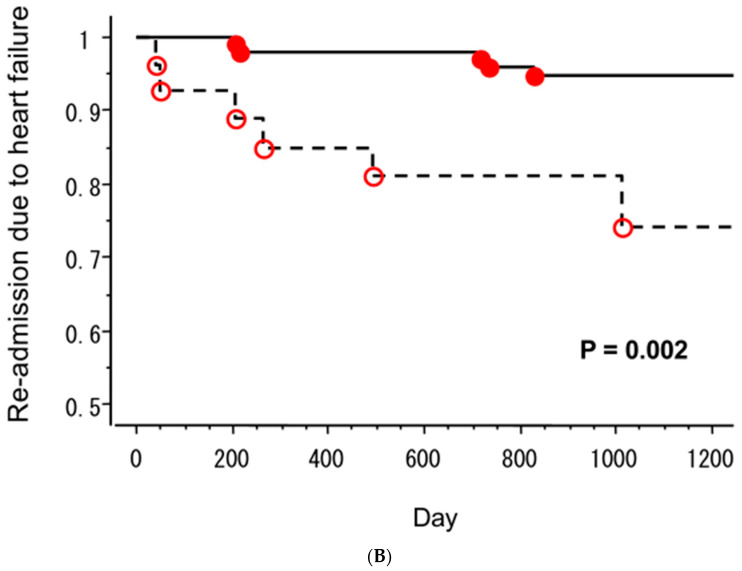
Long-term prognosis of the STEMI patients with and without hyponatremia 72 h after hospitalization. The Kaplan–Meier curve estimates the long-term occurrence of cardiac death (**A**) and rehospitalization due to heart failure (**B**). In patients with hyponatremia, the incidence of cardiac death showed a higher trend (**A**), and the incidence of re-admission due to worsening heart failure was significantly greater than that in the patients without hyponatremia (**B**). ●, patients with hyponatremia; ◯, patients with normonatremia 72 h after hospitalization. Cited from Ref. [5].

As mentioned above, early development of hyponatremia can occur during the early phase of ST elevation acute myocardial infarction (STEMI) [5,6,7,8] and could be an independent predictor of worsening heart failure during and after hospitalization. Singla et al. [8] showed that hyponatremia, serum Na less than 135 mmol/L, was linked to an increase in 30-day mortality and a recurrence of myocardial infarction in non-STEMI. Ploptowski et al. [7] demonstrated that low admission serum Na levels augmented the risk of in-hospital death or heart failure in STEMI. We also reported that the early development of hyponatremia increased the length of hospital stay and the incidence of in-hospital heart failure in STEMI [5]. Early development of hyponatremia could be a reliable predictor of long-term readmission due to heart failure. Goldberg et al. [6] reported that long-term mortality and rehospitalization due to heart failure increased in hyponatremic patients with STEMI. In comparison with previous studies, we showed that hyponatremia increased rehospitalization due to heart failure but was not associated with an increase in mortality rate (Figure 4). This difference might be explained by the successful percutaneous cardiac intervention in our study.

### 1.4. Treatment of Heart Failure with AVP V2 Receptor Antagonists

The use of AVP V2 receptor inhibitors to block the antidiuretic action of AVP may reduce circulatory blood volume in CHF. We previously demonstrated that a peptide AVP V2 receptor antagonist reduced water retention in the experimental rat model of fluid retention after inferior vena cava ligation [12]. Schrier et al. [13] showed that mozavaptan, a non-peptide AVP V2 receptor antagonist, suppressed AQP2 mRNA expression and water retention in an experimental model of heart failure in rats with ligated coronary arteries, suggesting that non-peptide AVP V2 receptor antagonists may effectively treat patients with heart failure. Several non-peptide AVP V2 receptor antagonists, such as mozavaptan, conivaptan, satavaptan, and tolvaptan, have been developed since 1992 [27,28]. These non-peptide AVP antagonists have potent and selective V2 receptor antagonism, and most can be used by oral administration. Tolvaptan is metabolized primarily by CYP3A. Tolvaptan, which can be administered orally and is metabolized by CYP3A, and conivaptan, which can only be administered intravenously, have been used to treat patients with hyponatremia and impaired water excretion. Tolvaptan selectively increases free water excretion in normal subjects and patients with CHF. Tolvaptan-induced aquaresis is not associated with natriuresis, kaliuresis, or changes in GFR. Tolvaptan has been used to treat the hyponatremia associated with the syndrome of inappropriate secretion of antidiuretic hormone (SIADH), CHF, and decompensated cirrhosis [29].

There have been many studies testing the efficacy of tolvaptan in CHF since 2000. These studies have had two main objectives: (1) to determine whether tolvaptan improved hyponatremia and reduced edema and total body Na and water and (2) to determine whether it improved or corrected the hyponatremia associated with CHF, cirrhosis of the liver, and SIADH in the United States and Europe [29]. Tolvaptan promptly increased serum Na levels during the initial 4 days, followed by a sustained elevation during the additional 30-day observation period. In the subsequent SALTWATER study, tolvaptan was continued for an additional 4 years in 111 patients who participated in the SALT study. The SALTWATER study clearly demonstrated that tolvaptan successfully kept serum Na levels within the normal range during the 4-year observation period [30].

Many studies have tested the effects of tolvaptan on a number of clinical outcomes since 2003. Studies carried out before 2010 have shown that tolvaptan improves short-term outcomes but does not affect long-term outcomes [2,31,32,33,34]. Gheorghiade et al. [31,32] reported that the oral administration of tolvaptan increased urine volume, decreased body weight, and increased serum Na levels, but it did not alter worsening heart failure during the 60-day observation period. Konstam et al. [33] studied the efficacy of tolvaptan in 4133 patients with CHF from 359 institutions in the United States (EVEREST study). The mean follow-up period was 9.9 months. In the short term, patients who had short-term greater water diuresis had a greater reduction in body weight and improved dyspnea, orthopnea, fatigue, and edema, and they had a shortened length of stay. In the long term, tolvaptan did not alter total and cardiovascular mortality rates. However, a subanalysis of the data verified a significant reduction in cardiovascular mortality by tolvaptan only in hyponatremic patients with serum Na levels of <130 mmol/L [33].

In Japan, tolvaptan has been available for the treatment of CHF since 2010 [35]. Tolvaptan acutely increased water diuresis and reduced fluid overload and promptly reduced congestion. Recent studies have addressed the long-term outcomes of treating CHF with tolvaptan. Uemura et al. [36] reported that tolvaptan decreased cardiac death and rehospitalization. Kinugawa et al. [37] reported that tolvaptan significantly improved the cardiac mortality rate in a study of 3349 patients with CHF. Similar results have been obtained by other investigators [38,39,40,41]. On the other hand, others have not shown any improvements in long-term outcomes by tolvaptan. Felker et al. [42] reported in the TACTICS-HF study that tolvaptan decreased the body weight of 257 patients with acute heart failure but did not show any differences in the length of hospital stay or post-discharge outcomes between the tolvaptan and placebo group. 

Wang et al. [43] performed a meta-analysis by selecting 14 of 22 studies. They found that tolvaptan significantly decreased body weight, increased urine volume and serum Na concentrations, and reduced dyspnea. However, they concluded that no dose of tolvaptan significantly reduced all-cause mortality or rehospitalization. Several reports from Japan have suggested that tolvaptan improves some long-term outcomes, but the majority of studies have failed to show any improvements in long-term outcomes for mortality and rehospitalization. It appears that the major clinical benefits of AVP V2 receptor antagonists are related to the consistent long-term correction of hyponatremia, which would decrease the hyponatremia-induced five-fold increase in falls and bone fractures [44,45]. The most important clinical utilization of tolvaptan is to correct hyponatremia and reduce or eliminate the falls and bone fractures that are associated with serum Na levels less than 132 mmol/L [44,45]. Tolvaptan should start at a dose of 15 mg, and the dose should be titrated to higher levels if there is little or no increase in urine output or improvement in serum Na levels. The most important caution is to prevent an increase in serum Na by more than 6 mmol/L over a 24 h period to reduce the possibility of developing osmotic demyelination [46]. Serum Na levels should be monitored more frequently when urine output increases significantly. The rate of increasing serum Na can be retarded by infusing 5% dextrose in water or administering intranasal dDAVP.

By contrast, the long-term increases in mortality, rehospitalization, and persistent CHF appear to be related to volume overload due to the renal retention of Na and water and not due to an increase in water alone. As discussed in a manuscript in this special issue, the recently identified natriuretic protein, haptoglobin-related protein without signal peptide (HPRWSP), from the sera of patients with renal salt wasting satisfies our long search for a potent proximal diuretic [47,48]. It will be interesting to determine whether the combination of the newly identified proximal natriuretic protein HPRWSP with conventional distal diuretics, such as furosemide, can eliminate all or most of the fluid overload that appears to be the independent predictor of long-term mortality rates, recurrence of CHF, and rehospitalization.

## 2. Conclusions

The present review indicates that hyponatremia is frequently found in patients with CHF who have a weakened myocardium, which leads to a complicated set of pathophysiologic circumstances. The weakened myocardium reduces cardiac output, which sequentially decreases the effective circulating volume; increases AVP production via baroreceptor-mediated non-osmotic stimuli; and increases renal water reabsorption, which induces hyponatremia. The increase in renal Na and water reabsorption increases total body Na and water and edema formation through alterations in the sympathetic nervous system and increases in plasma renin, angiotensin, and aldosterone levels, and it activates complicated hemodynamic factors. In addition, hyponatremia predicts worsening clinical outcomes in patients with CHF. The predictable correction of hyponatremia by the utilization of AVP V2 receptor antagonists will ameliorate the frequency of falls and bone fractures associated with hyponatremia. Finally, it will be interesting to determine whether the combination of the newly identified potent proximal natriuretic protein HPRWSP with conventional distal diuretics eliminates or partially eliminates the fluid overload that appears to be an independent predictor of long-term mortality, recurrence of CHF, and rehospitalization.

## Data Availability

The data used to the finding of this study are available from the corresponding author upon request.
